# A combination of mutations in *AKR1D1* and *SKIV2L* in a family with severe infantile liver disease

**DOI:** 10.1186/1750-1172-8-74

**Published:** 2013-05-16

**Authors:** Neil V Morgan, Jane L Hartley, Kenneth DR Setchell, Michael A Simpson, Rachel Brown, Louise Tee, Sian Kirkham, Shanaz Pasha, Richard C Trembath, Eamonn R Maher, Paul Gissen, Deirdre A Kelly

**Affiliations:** 1 Department of Medical and Molecular Genetics and Centre for Rare Diseases and Personalised Medicine, University of Birmingham School of Medicine, Birmingham, UK; 2 Liver Unit, Birmingham Children’s Hospital, Steelhouse Lane, Birmingham, UK; 3 Department of Pathology and Laboratory Medicine, Cincinnati Children’s Hospital Medical Center and Department of Pediatrics, University of Cincinnati College of Medicine, Cincinnati, OH, 45229, USA; 4 Medical and Molecular Genetics, King’s College London, 8th Floor Tower Wing, Guy’s Hospital, Great Maze Pond, London, UK; 5 Department of Cellular Pathology, Queen Elizabeth Hospital Birmingham, University Hospitals Birmingham NHS Foundation Trust, Mindelsohn Way, Edgbaston, Birmingham, UK; 6 Department of Paediatric Gastroenterology, Queens Medical Centre, Nottingham, UK; 7 West Midlands Regional Genetics Service, Birmingham Women’s Hospital, Birmingham, UK; 8 UCL Institute of Child Health and Laboratory for Molecular Cell Biology, London, UK; 9 Present address: Centre for Cardiovascular Sciences, Institute of Biomedical Research, College of Medical and Dental Sciences, University of Birmingham, Birmingham B15 2TT, UK

**Keywords:** Bile acid metabolism, Diarrhoea, Gene mutation, Whole exome sequencing, Paediatric liver disease

## Abstract

Infantile cholestatic diseases can be caused by mutations in a number of genes involved in different hepatocyte molecular pathways. Whilst some of the essential pathways have a well understood function, such as bile biosynthesis and transport, the role of the others is not known. Here we report the findings of a clinical, biochemical and molecular study of a family with three patients affected with a severe infantile cholestatic disease. A novel homozygous frameshift germline mutation (c.587delG) in the *AKR1D1* gene; which encodes the enzyme Δ ^4^-3-oxosteroid 5β–reductase that is required for synthesis of primary bile acids and is crucial for establishment of normal bile flow, was found in all 3 patients. Although the initial bile acid analysis was inconclusive, subsequent testing confirmed the diagnosis of a bile acid biogenesis disorder. An additional novel homozygous frameshift mutation (c.3391delC) was detected in *SKIV2L* in one of the patients. *SKIV2L* encodes a homologue of a yeast ski2 protein proposed to be involved in RNA processing and mutations in *SKIV2L* were recently described in patients with Tricohepatoenteric syndrome (THES). A combination of autozygosity mapping and whole-exome-sequencing allowed the identification of causal mutations in this family with a complex liver phenotype. Although the initial 2 affected cousins died in the first year of life, accurate diagnosis and management of the youngest patient led to successful treatment of the liver disease and disease-free survival.

## Findings

### Background

Neonatal cholestasis affects approximately one in 2,500 births and results from diminished bile flow or secretion. The aetiology of the neonatal cholestasis is highly variable and includes infections, inborn errors of metabolism including bile acid synthesis and transport disorders, and mechanical obstruction (e.g. biliary atresia) [1]. Early recognition and diagnosis of neonatal cholestasis is essential to permit effective clinical management. In recent years new genetic causes of cholestasis have been defined which have led to a greater understanding of the mechanisms of bile secretion, liver development and physiology. These include among others, defects in bile acid synthesis [2], and transport. However, the function of many genes that are associated with cholestatic diseases is still poorly understood. Recent advances in gene sequencing technologies have made it possible to determine the sequence of most genes, and these novel approaches facilitate identification of disease causing mutations in patients with complex phenotypes [3].

### Case reports

**Figure 1:**
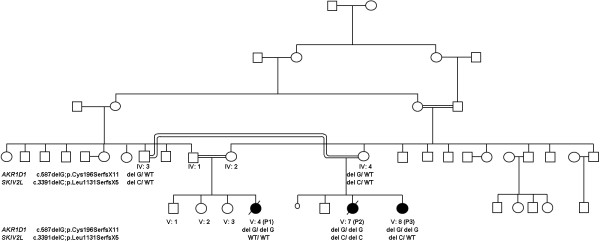
**Identification of *AKR1D1 *and *SKIV2L *mutations in a multiple-consanguineous family with severe infantile liver disease.** The initial proband investigated is patient 1 (V:4; P1) her cousin (V:7; P2) is patient 2 and affected sibling (V:8; P3). Solid symbols represent affected individuals. The segregation of the 2 mutations identified are shown. The upper mutation is a homozygous single base G deletion in *AKR1D1 *leading to a frameshift and premature stop codon (c.587delG; p.Cys196SerfsX11) and lower mutation a single base C deletion in *SKIV2L *leading to a frameshift and premature stop codon (c.3391delC;p.Leu1131SerfsX5).

We studied a consanguineous family of Pakistani descent consisting of three patients who were first cousins (Figure 1).

#### Patient 1

**Figure 2:**
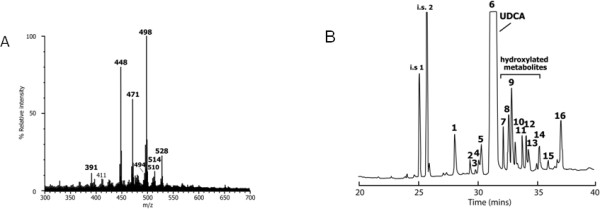
**Mass Spectrometric analysis of Urine. **(**A**) The negative ion fast atom bombardment ionization mass spectrum of the urine from patient 1. (**B**) GC-MS analysis of the methyl ester-trimethylsilyl ether derivatives of bile acids in the urine of patient 1 identified with a mutation in the *AKR1D1 *gene and undergoing bile acid therapy with ursodeoxycholic acid. The following compounds were identified from their mass spectra and retention indices: 1. cholesterol; 2. 3a,7a,12a-trihydroxy-5a-cholanoic and 3b-hydroxy-5-cholenoic acids; 3. unknown; 4. unknown; 5. 3a,6a-dihydroxy-5b-cholanoic; 6. 3a,7b-dihydroxy-5b-cholanoic (UDCA); 7. 3,7-dihydroxy-cholanoic isomer; 8. 3-oxo-7a-hydroxy-4-cholenoic; 9. oxo-dihydroxy-cholanoic; 10. trihydroxy-cholanoic; 11. impurity; 12; dihydroxy-cholanoic acid; 13. 3-oxo-7a,12a-dihydroxy-4-cholenoic; 14. 1b,3a,7a-trihydroxy-cholanoic; 15. 3a,6b,7b-trihydroxy-5b-cholanoic; 16. 3a,4b,12a-trihydroxy-cholanoic; i.s. 1. internal standard, coprostanol; i.s. 2 internal standard, nordeoxycholic acid.

The proband, (*Patient 1,* V:4), a female child was born at full-term by Caesarean section and was well and breast-fed for one month before switching to standard infant formula. At nine weeks of age she was investigated for progressive cholestasis (serum bilirubin 534 μmol/L (0-15 μmol/L), gamma glutamyl transferase (GGT) 131 IU/L (25-70 IU/L), alanine transaminase (ALT) 1769 IU/L (5-45 IU/L), aspartate transaminase (AST) 2078 IU/L (20-60 IU/L)) and persistent hypoglycaemia. She had no dysmorphic features and no diarrhoea. She was found to be hyperinsulinaemic and required diazoxide to establish good glycaemic control. At nine weeks of age she had a firm liver, which was palpable 3 cm below the costal margin and no splenomegaly. An abdominal ultrasound scan, chest x-ray and ocular examination were all entirely normal. A radioisotope scan of bile secretion (TiBIDA) identified good uptake of isotope into the liver but no excretion. Cardiac and ophthalmology investigations were all normal. Metabolic screen was consistent with a liver dysfunction but excluded metabolic causes of cholestasis, such as galactosaemia, tyrosinaemia, cystic fibrosis, amino and organic acid disorders, glycosylation defects, glycogen storage diseases, mitochondrial respiratory chain and fatty acid oxidation defects. The negative ion FAB-MS analysis (Additional file 1: supplementary methods) of the urine revealed dominant ions at m/z 448 and 498 that were consistent with glycine and taurine conjugated dihydroxy-cholanoic acids, respectively. These ions are consistent in mass to the normal primary bile acids conjugates of chenodeoxycholic acid, suggesting that there was no defect in primary bile acid synthesis (Figure 2). Similarly, the low intensity ions at m/z 464 and m/z 514 indicated the presence of the corresponding trihydroxy-cholanoic acid conjugates, which would be consistent with cholic acid conjugates. There were low intensity ions at m/z 494 and 510, which reflected the presence of oxo-monohydroxy- and oxo-dihydroxy-cholenoic acids (unsaturated bile acids). In addition, the ions at m/z 471 and m/z 528 were consistent with the presence of sulfate and glyco-sulfate conjugates of dihydroxy-cholanoic acids these ions are typically present in the mass spectra of urine from patients undergoing ursodeoxycholic acid (UDCA) treatment.

A liver biopsy at 2 months of age showed florid giant-cell transformation, marked cholestasis and ballooned hepatocytes. There were numerous areas of extramedullary haematopoesis and mild iron deposition. There was no mature fibrosis and ductular proliferation was rarely seen. Electron microscopy showed whorled inclusions and myelin figures as well as large pale inclusions containing electron dense material the nature of which was uncertain. She was diagnosed with neonatal hepatitis due to a possible disorder of bile transport and treated with ursodeoxycholic acid and supportive nutrition including fat-soluble vitamins. Despite this her liver function deteriorated (prothrombin time 28 seconds, albumin 21 g/L) and she developed ascites. She was listed for liver transplantation but died awaiting a suitable organ.

#### Patient 2

*Patient 2* (V:7), a female cousin of patient 1 was born at term by Caesarean section for symmetrical intrauterine growth retardation weighing 1770 g (<0.4th centile) and reduced liquor. She was well at birth but soon developed hypoglycaemia, which required intravenous infusions of high concentration (18 mg/kg/min) dextrose solution.

She had a rising conjugated hyperbilirubinaemia from birth (285 μmol/l at 6 months) with normal GGT 67 μmol/L. Other transaminases were abnormally raised (ALT 441 IU/L, AST 84 IU/L). Liver biopsy at six months showed marked cholestasis and hepatocyte ballooning with giant cell transformation. There was fibrous expansion of the portal tracts with localized bridging fibrosis.

Within two weeks of birth she also developed severe life threatening diarrhoea with watery yellow stool, requiring parenteral nutrition from four weeks of age to maintain growth and hydration. At the time of hypoglycaemia, growth hormone, cortisol and lactate were normal. Although a suppressed ketotic response was found, there was no recorded hyperinsulinism. The stool investigations showed a predominant chloride loss (102), with sodium 90 mEq/L, osmolarity 244 mosmol/kg, pH 7.9 and normal faecal elastase. Common causes of cholestasis were excluded as in patient 1. Bone marrow examination was normal.

Duodenal biopsies showed subtotal villous atrophy with an increased number of intraepithelial lymphocytes and epithelial apoptosis. There were no histological features suggestive of microvillous inclusion disease, tufting enteropathy or autoimmune enteropathy. The serum immunoglobulin levels were low (IgG < 0.01 g/L (0.3-0.5). Investigation of the patient’s hair identified trichorrhexis nodosa with low cysteine and high leucine content, which led to the consideration of THES as the underlying unifying diagnosis. However, sequencing of the exons and exon-intron boundaries was negative for mutations in *TTC37*.

The liver disease progressed rapidly with serum bilirubin rising to 389 μmol/L, falling albumin (20 g/L (34-42 g/L), rising prothrombin time (21 seconds (normal range 10-13 seconds) and falling platelets (40 ×10^9^/L (150-400 ×10^9^/L). Liver transplantation was proposed but the family requested active palliation with death occurred at the age of 9 months.

### Molecular Genetic analysis and identification of *AKR1D1* novel mutation

As the known causes of cholestatic liver disease were excluded in this family we hypothesized that all affected children had a novel autosomal recessively inherited disorder and undertook genetic linkage studies in combination with whole exome sequencing on the genomic DNA of affected patients 1 and 2. Genome-wide SNP genotyping using the Affymetrix 250 k SNP microarray and analysis using HomozygosityMapper in both affected children showed the largest overlapping autozygous regions at chromosome 7 (134,406,000-159,126,632 bp), chromosome 16 (906,887-11,568,826 bp), chromosome 20 (55,452,732-59,208,834 bp), chromosome 12 (64,283,014-67,448,660 bp) and chromosome 4 (77,794,236-79,095,407 bp). In addition the exomes of patients 1 and 2 were sequenced (Additional file 1: supplementary methods) and the alignment of the sequencing reads revealed that 83% and 76% of the CCDS defined exome was covered by >20 high quality reads identifying 17,844 and 17,867 variations in patients 1 and 2 respectively. Comparisons with dbSNP build 131, the 1000 Genomes project database and our in-house database (composed of 40 exomes), identified 7, 6, 1, 1 and 1 novel variants in the chromosome 7, 16, 20, 12 and 4 candidate regions respectively. Of these only 3 homozygous nonsynonymous variants and 1 frameshift variant were found in both patients. The frameshift was a homozygous single base G deletion (c.587delG) in exon 6 of *AKR1D1* that mapped within the chromosome 7 candidate linkage region of the family. The variant results in a frameshift at amino acid 196 leading to a premature stop codon 11 amino acids downstream (p.Cys196SerfsX11). We validated this variant by Sanger sequencing on genomic DNA, which segregated with the disease status in all family members (Figure 1). *AKR1D1* encodes Δ^4^-3-oxosteroid 5β-reductase, however the initial bile acid analysis in patient 1 seemed to exclude a bile acid biosynthesis defect.

### Successful treatment of the new Patient 3 with cholic acid

A female sibling (*Patient 3*; V:8) of Patient 2, was born following a normal pregnancy by Caesarean section at term with a birth weight of 7 3.4 kg. She developed neonatal jaundice, which cleared spontaneously and was reviewed at five weeks of age. DNA from this patient was screened for the presence of the *AKR1D1* mutation that was identified in the other 2 affected children in this family and indeed was found to be homozygous for c.587delG.

A urine sample was obtained from patient 3 and analyzed by the same method described above. The negative ion FAB-MS spectrum profile indicated a marked elevation in urinary bile acid excretion consistent with a severe cholestasis. The profile revealed elevations in taurine and glycine conjugates of unsaturated oxo-dihydroxy and oxo-trihydroxy bile acids. Intense ions at m/z 460 and 510 reflected the presence of high concentrations of Δ^4^-3-oxo bile acids that are characteristic of a bile acid synthetic defect involving a deficiency in the activity of Δ^4^-3-oxosteroid 5β-reductase enzyme. The child was initially started on 15 mg/kg/day of Cholic Acid in three divided doses and the liver function tests remained normal until an episode of non-compliance with medication when the following abnormalities were identified: ALT of 390 IU/L, AST 324 IU/L and GGT 40 IU/L but normal bilirubin of 6 mmol/L. Liver function became normal again at the age of 5 months after improved compliance with the medication. The child was well, asymptomatic for presence of liver disease and developing normally at the time of the most recent review aged 12 months.

### Identification of an additional *SKIV2L* mutation in patient 2

The striking clinical THES phenotype in addition to the liver disease identified in patient 2 and absence of it in her cousin, who had proven Δ^4^-3-oxosteroid 5β-reductase deficiency, suggested that additional pathology was present in patient 2 compared with her sibling (patient 3) and cousin (patient 1). More recently *SKIV2L* mutations have been described in THES patients negative for *TTC37* mutations [4]. We then scrutinized the whole exome data in patient 2 and specifically looked for mutations present in patient 2 which were absent in patient 1. This revealed a homozygous frameshift mutation in *SKIV2L* (c.3391delC; p.Leu1131SerfsX5) in patient 2 which was not present in her affected sibling and cousin (Figure 1).

## Conclusions

We have described three patients from a multiple consanguineous family with severe liver disease. In addition, patient 2 had severe diarrhoea requiring parenteral nutrition, and other features of THES including hair amino acid abnormalities which are found in these patients. As part of the clinical investigations sequencing of *TTC37*, mutations in which were known to cause THES, was undertaken but revealed no abnormalities. A combination of genome wide linkage scan and whole exome sequencing was performed to look for other genetic causes of liver disease in this family. All three of the patients were found to have a homozygous mutation in *AKR1D1*, consistent with a treatable bile acid biosynthesis disorder. Further bile acid analysis and successful treatment of patient 3 confirmed the initial diagnosis. Furthermore, patient 2 was found to have an additional disease causing mutation in *SKIV2L*, that was only recently described to cause THES.

Bile acid synthesis disorders can normally be identified by measurement of bile acids in the urine using liquid secondary ionization mass spectrometry [2]. However, the presence of exogenous bile acids, such as ursodeoxycholic acid (UDCA), may mask the ability to detect a bile acid synthetic defect because UDCA and primary bile acids, being stereoisomers, have the same molecular weight. The FAB-MS analysis of the urine from patient 1, performed in 2003, revealed very high concentrations of dihydroxy-bile acid conjugates derived from the administered UDCA. There was no evidence for substantial amounts of Δ^4^-3-oxo bile acids that characterize a deficiency in Δ^4^-3-oxosteroid 5β-reductase [5], and the mass spectrum was not consistent with this bile acid disorder [2]. More comprehensive GC-MS analysis performed 8 years later on this original urine sample, and only after the identification of the mutation in *AKR1D1*, showed that Δ^4^-3-oxo bile acids, while present, accounted for <2% of the total bile acids in urine, and the 5α-reduced metabolite, allo-cholic acid also synthesized in patients with Δ^4^-3-oxosteroid 5β-reductase deficiency was also found in traces [6], because the urine was dominated by UDCA and its metabolites (data not shown). Δ^4^-3-Oxo bile acids are normal metabolites of the urine of neonates and are consistently found in cases of advanced liver disease [7-10]. Cholic or chenodeoxycholic acid are normally also detected in the urine however in our case they were not identified. 5β-reduced bile acids were identified in the urine of patient 1 which are presumed to be metabolites of UDCA (a 5β-cholanoic acid) and its metabolites. Thus, the more comprehensive GC-MS analysis of the urine of patient 1 would support the diagnosis of a Δ^4^-3-oxosteroid 5β-reductase deficiency, with the complementary molecular confirmation of a mutation in *AKR1D1* in this patient.

Bile acid synthesis disorders account for approximately 2% of idiopathic forms of liver disease but are important to diagnose as they can be treated by supplementation of primary bile acids, which is highlighted by this report. Indeed the new affected sibling of Patient 2 responded well to treatment with Cholic acid.

In conclusion we have extended the clinical features of Δ^4^-3-oxosteroid 5β-reductase enzyme deficiency as severe hypoglycaemia has not been previously reported. We also highlight the importance of performing urinary bile acid analysis in the absence of UDCA therapy in combination with genetic analysis. We also expand the mutational spectrum of *SKIV2L* and provide the first report of a combined phenotype of THES and Δ^4^-3-oxosteroid 5β-reductase enzyme deficiency. Combining the technique of whole genome linkage mapping and whole exome sequencing creates a powerful tool to elucidate the molecular basis of uncharacterized genetic disorders.

### Patient consent and ethics approval

This study was conducted according to the principles expressed in the Declaration of Helsinki. The study was approved by the local Research Ethics Committees. All participants provided written informed consent for the collection of samples and subsequent analysis. In the case of children, consent was obtained from the patient’s guardian, parent or next of kin.

## Competing interests

The authors have no relevant conflicts of interests to declare.

## Authors’ contributions

NM and MAS performed experimental work, interpreted data and drafted and revised the manuscript. JLH recruited patients to the study, performed experimental work, interpreted data and drafted and revised the manuscript. KDRS, RB and LT performed the experimental work, interpreted data and revised the manuscript. SK and SP referred the patient to the study team and revised the manuscript. RCT and ERM interpreted data and revised the manuscript. PG and DAK recruited patients to the study, interpreted data and drafted and revised the manuscript. All authors read and approved the final manuscript.

## Supplementary Material

Additional file 1 **Supplementary methods [**[11]**].** Click here for file
