# Supplementary material for: A combination of mutations in AKR1D1 and SKIV2L in a family with severe infantile liver disease
Source: Orphanet J Rare Dis. 2013 May 16;8:74. doi: 10.1186/1750-1172-8-74 (PMC3659031; doi:10.1186/1750-1172-8-74)
Supplement: Additional file 1 — Supplementary methods [[11]]. [file 1750-1172-8-74-S1.doc]

**Supplementary methods**

**Whole exome Sequencing**

In parallel to the linkage analysis, we sequenced the whole exome of patients 1 and 2 with the SureSelect human All Exon kit (Agilent Technologies) and sequencing on the Illumina GaIIx with 76bp paired-end reads. The sequences obtained were aligned to the reference genome (hg18 build), with Novoalign (Novocraft Technologies Sdn Bhd). Duplicate reads, resulting from PCR clonality or optical duplicates, and reads mapping to multiple locations were excluded from downstream analysis. Depth and breadth of sequence coverage was calculated with custom scripts and the BedTools package. Single nucleotide substitutions and small insertion deletions were identified and quality filtered within the SamTools software package and in-house software tools. All calls with a read coverage <4x and a phred-scaled SNP quality of <20 were filtered out. Variants were annotated with respect to genes and transcripts with the VariantClassifier tool. Filtering of variants for novelty was performed by comparison to dbSNP131 and 1000 Genomes pilot SNP calls (March 2010) and variants identified in 40 control exomes sequenced and analysed by the same method described above.

**Detailed Mass Spectrometry analysis**

Negative ion FAB-MS spectra were recorded after placing the equivalent of 0.01 mL of the urine onto a small drop of glycerol spotted on the target probe. The probe was introduced directly into the ion source of Waters Autospec magnetic sector mass spectrometer and a beam of fast atoms of cesium was fired at the target containing the sample. Negative ion spectra (scan time 1.5 s) were recorded over the mass range (m/z) 50-800 Da. A list of ions relevant to bile acid conjugates has been published previously and features of the mass spectra characterizing all of the bile acid synthetic defects identified thus far have been previously published

The same urine extract was further analyzed to determine the qualitative and quantitative pattern of bile acid excretion by gas chromatography-mass spectrometry after solvolysis and hydrolysis of the conjugates, re-extraction, separation from neutral sterols, and preparation of the volatile methyl ester-trimethylsilyl (Me-TMS) ether derivatives. These derivatives were separated by chromatography on a 30 m X 0.4-mm DB-1 (film thickness 0.25 mm) fused silica capillary column (J and W Scientific, Folsom, CA) operated in temperature programming mode from 225º - 295ºC with increments of 2ºC/min, and initial and final isothermal periods of 5 and 20 min, respectively. Helium was used as carrier gas at a flow rate of 1.8 ml/min. GC-MS was carried out on an Autospec magnetic sector GC-MS instruments housing identical GC columns and operated under the same gas chromatographic conditions. Electron ionization (70 eV) mass spectra were recorded over the mass range 50-800 Da by repetitive scanning (1.5 s/cycle) of the eluting components of the complete GC profile. Bile acids were quantified by gas chromatography from a comparison of their peak area response with the peak area response obtained for the known amount of the added internal standard, nordeoxycholic acid added to the sample prior to sample preparation. The identification of a bile acid was based upon its GC retention index relative to a homologous series of n-alkanes (MU value) and the EI mass spectrum compared with a published library of reference bile acids or from known fragmentation patterns
